# What forms of framing can be found in the Flemish media in a jury trial about euthanasia for psychological suffering?

**DOI:** 10.1192/j.eurpsy.2025.193

**Published:** 2025-08-26

**Authors:** K. Catthoor

**Affiliations:** 1 CAPRI, University of Antwerp; 2 PZ Stuivenberg, Ziekenhuis aan de Stroom, Antwerp; 3 Flemish Association of Psychiatry, Kortenberg, Belgium

## Abstract

**Abstract:**

The Flemish Association of Psychiatry developed due care guidelines for medical assisted dying in cases of severe and unbearable psychiatric suffering, which were adopted by the Order of Physicians. In principle, there are therefore more than enough well-known recommendations on how to handle a request for termination of life from a patient with psychiatric issues.

Nevertheless, occasionally something goes wrong due to misinterpretation of the legal criteria or due to careless actions by the consulting or performing physician.

In 2010, a female patient died by euthanasia because of unbearable mental suffering, which was unacceptable for her family. The family decided to initiate a court case to have the inaccuracies in the decision-making process and the execution of the euthanasia evaluated by a judge. In 2020, three involved doctors, including a psychiatrist, were prosecuted for this euthanasia. An analysis of the court case and the media coverage of this case will be discussed.

**Disclosure of Interest:**

None Declared

